# Reduced Emergency Department Utilization after Increased Access to Primary Care

**DOI:** 10.1371/journal.pmed.1002114

**Published:** 2016-09-06

**Authors:** Sanjay Basu, Russell S. Phillips

**Affiliations:** 1 Department of Medicine, Stanford University, Stanford, California, United States of America; 2 Center for Primary Care, Harvard Medical School, Boston, Massachusetts, United States of America; 3 Division of General Medicine and Primary Care, Beth Israel Deaconess Medical Center, Boston, Massachusetts, United States of America

## Abstract

Sanjay Basu and Russell Phillips discuss the findings from Whittaker and colleagues on the link between extending primary care hours and emergency department utilization.

Reducing emergency department utilization by increasing access to primary care has been a long-standing quest. In 1958, physicians at Hartford Hospital, Connecticut, lamented in *The New England Journal of Medicine* that only a minority of emergency department utilization could be attributable to medical conditions warranting inpatient hospital admission; among hospitals’ administrators surveyed by the authors, most wanted to reduce emergency department utilization by increasing access to ambulatory services, “exclud[ing] the cases that could be handled in a doctor’s office,” or instructing patients to first contact their family physician to perform triage [1].

The effort to reduce emergency department utilization for “ambulatory-sensitive conditions” has become increasingly politically contentious in the United Kingdom, as the National Health Service has been reformed to reduce central government control, in turn increasing fragmentation and complexity of the system [2]. In this context, over one-quarter of unplanned emergency department visits followed unsuccessful attempts to access primary care [3]. Access to primary care is even more challenging among low-income patients in the United States; a recent US Inspector General’s investigation into managed Medicaid programs (insurance for very low-income adults) reported that more than half of providers could not actually offer enrollees appointments—given unwillingness to accept their insurance and backlogs of appointments—and those who could offer an appointment had a median wait time of two weeks, with a quarter having a wait time of over one month [4].

## The Inadequacy of Prior Research on a Common Goal

Surprisingly few studies have carefully examined whether emergency department utilization has been reduced by increasing access to primary care. Per a 2013 systematic review of international studies, most research on the question has been poorly designed [5]. Many prior studies simply correlated differences in emergency department utilization with differences in primary care access—a study design confounded by differences in patient composition between low- and high-access groups. Other studies compared emergency department utilization among patients before versus after interventions to increase primary care access, which makes the results highly subject to confounding by external influences, such as changes in the economy (as unemployment affects care-seeking) or even seasonality (e.g., increased utilization in influenza season).

In this issue of *PLOS Medicine*, William Whittaker and colleagues provide a better-designed study of a natural experiment in the UK, in which some practices in 2014 expanded primary care access by enhancing service hours [6]. The practices offered urgent care appointments on weekday evenings as well as on both weekend days. Whittaker and colleagues not only compared the participating practices to a control group offering standard access but also addressed a limitation in the field—an assumption of the common "difference-in-differences" method utilized to study natural experiments. The difference-in-difference method compares patients in the "treatment" (primary care expansion) practices to those in the "control" (usual care) practices before and after the policy change to account for time trends and other factors that might similarly affect both sets of patients (e.g., the economy or seasonality) [7]. Yet the difference-in-differences method requires that we assume treatment and control groups would not systematically differ over time in ways related to trends in emergency department utilization, except for the influence of the expansion in primary care access. This assumption is problematic; the treatment group practices volunteered to expand service hours, perhaps reflecting an organizational culture emphasizing attentive patient care. To account for differences among the treatment and control practices, Whittaker and colleagues matched similar practices in both the treatment and control groups, then performed their difference-in-differences analysis on a well-matched sample. Combining matching with difference-in-differences analysis has become increasingly popular [8], as an “ideal” control group is rarely found; newer "synthetic control" methods to construct a well-matched control group from imperfect options will likely facilitate more studies of natural experiments in the future [9,10]. Yet, the matching-based approach may not fully control for unmeasured factors (e.g., the culture of a practice) that could still bias the results.

Nevertheless, Whittaker and colleagues observed a 26.4% reduction in patient-initiated referrals to emergency departments for "minor" problems among patients with increased access to primary care, as compared to their counterparts in the control group.

## Headlines and Subtler Lessons

For health services researchers, the most informative results may be found in the authors’ careful process evaluation. Providing enhanced primary care access was costly and produced a heavy workload. An average of 35 additional hours of appointments per week were made available per practice in the treatment group, resulting in ~33,000 additional primary care appointments booked at primary care practices and US$4.8 million in costs. By contrast, about 11,000 emergency department visits were averted, which would have cost ~US$1.1 million. Hence, expanding access to primary care did not result in a cost savings. The intervention may still be cost-effective, which requires longer-term data on health outcomes.

In an era in which provider satisfaction has been added to the oft-cited “triple aim” of improving health, improving quality of care, and reducing costs, a concern posed by the results is whether narrowly focusing on emergency department utilization has superseded the quest to increase access to primary care in a manner that can be well-maintained into the future despite a heavy workload and time investment for primary care teams. While expanding access to primary care has become a major focus of many international efforts (such as the effort to achieve “patient centered medical homes” in the US or broader universal primary care access internationally), expanding access by including evening or weekend hours comes at a price, requiring additional information on the long-term impact of these expanded hours. In some regions, frequent users or “super-users” of emergency departments require far more services than simply providing evening or weekend hours to avert unnecessary emergency department utilization.

Whittaker and colleagues carefully catalogue these issues and provide support to a theory that emergency department utilization can be reduced through increased access to primary care—a contention commonly suggested in the past [11,12] but now having the rigor of a carefully controlled, large-scale natural experiment.
